# Molecular topography of the *MED12*-deleted region in smooth muscle tumors: a possible link between non-B DNA structures and hypermutability

**DOI:** 10.1186/1755-8166-6-23

**Published:** 2013-06-05

**Authors:** Dominique Nadine Markowski, Rolf Nimzyk, Gazanfer Belge, Thomas Löning, Burkhard Maria Helmke, Jörn Bullerdiek

**Affiliations:** 1 Center of Human Genetics, University of Bremen, Leobener Strasse ZHG, Bremen, D-28359, Germany; 2 Albertinen-Pathologie, Fangdieckstraße 75a, Hamburg, D-22547, Germany; 3 Institute of Pathology, University of Heidelberg, Im Neuenheimer Feld 220/221, Heidelberg, D-69120, Germany; 4 Present address: Institute of Pathology, Elbe Kliniken, Klinikum Stade, Bremervörder Str. 111, Stade, D-21682, Germany

**Keywords:** Deletions, Smooth muscle tumors, *MED12*, Non-canonical DNA-structures

## Abstract

**Background:**

Deletions of the gene encoding mediator subcomplex 12 (MED12) in human smooth muscle tumors rank among the most frequent genomic alterations in human tumors at all. In a minority of these cases, small deletions are found. In an attempt to delineate key features of the deletions aimed at a better understanding of the molecular pathogenesis of uterine smooth muscle tumors we have analyzed 70 *MED12* deletions including 46 cases from the literature and 24 own unpublished cases.

**Results:**

The average length of the deletions was 18.7 bp ranging between 2 bp and 43 bp. While in general multitudes of 3 clearly dominated leaving the transcript in frame, deletions of 21, 24, 30, and 33 nucleotides were clearly underrepresented. Within the DNA segment affected deletion breakpoints were not randomly distributed. Most breakpoints clustered within the center of the segment where two peaks of breakpoint clusters could be distinguished. Interestingly, one of these clusters coincides with the loop of a putative folded non-B DNA structure whereas a much lower number of breaks noted in the 5′ and 3′ stem of the structure forming an intramolecular B-helix. The second cluster mainly consisting of 3′ breaks was located in a region downstream adjacent to the stem.

**Conclusion:**

The present study describes for the first time main characteristics of *MED12* deletions occurring in smooth muscle tumors. Interestingly, the non-random distribution of breakpoints within the deletion hotspot region may point to a role of non-canonical DNA structures for the occurrence of these mutations and the molecular pathogenesis of uterine smooth muscle tumors, respectively.

## Background

The high prevalence of uterine fibroids (syn.: leiomyomata) not only makes them the most frequent symptomatic human tumors at all but also places recurrent mutations in these benign smooth muscle tumors among the most predominant mutations during tumorigenesis. Of the latter, rearrangements of the gene encoding high mobility AT-hook 2 protein (*HMGA2*) are accompanied by chromosomal translocations affecting its locus on chromosome 12 [1] and thus, as a rule, can be detected by classical cytogenetics. As a molecular consequence, HMGA2 becomes strongly upregulated [2,3]. Another, even more frequent type of mutations usually does not coincide with microscopically visible chromosomal deviations. Quite recently, mutations of *mediator subcomplex 12* (*MED12*), have been described in a majority of fibroids [4]. They mostly occur in those fibroids not displaying cytogenetic deviations but can also be associated e.g. with deletions of the long arm of chromosome 7 or chromosomal translocations involving 6p21 [5]. It almost goes without saying that due to the high prevalence of fibroids *MED12* mutations can be considered being the most frequent mutations in human tumors at all. They cluster within a hotspot region in the second exon of the gene with most of the mutations constituting single base exchanges within one triplett. Nevertheless, the same region is also often affected by in frame deletions. We feel that an in depth analysis of these deletions may contribute to a better understanding of the molecular pathogenesis of fibroids. Hence, we have summarized the data on *MED12* deletions from the cases published so far as well as from our own unpublished cases. In summary, a total of 70 smooth muscle tumors with *MED12* deletions including 46 cases published previously [4-11] as well as 24 own unpublished cases have been analyzed.

## Results

A total of 70 smooth muscle tumors with *MED12* deletions retrieved from the literature (46 cases), as well as from further unpublished own cases (24 cases) were analyzed (Table 1).

**Table 1:** **Details of 70 *MED12 *deletions analyzed**

**Diagnosis/Location**	**Deletion**	**Length (bp) of the deletion**	**Reference**
Uterine Leiomyoma	c.117_152del	36	[8]
Uterine Leiomyoma	c.118_159del	42	[8]
Uterine Leiomyoma	c.121_135del	15	[8]
Uterine Leiomyoma	c.131_145del	15	[8]
Uterine Leiomyoma	c.137_151del	15	[8]
Uterine Leiomyoma	c.136_153del	18	[8]
Uterine Leiomyoma	c.121_132del	12	[8]
Uterine Leiomyoma	c.124_137del	14	[8]
Uterine Leiomyoma	c.149_163del15	15	[6]
Uterine Leiomyoma	c.122_148del27	27	[6]
Uterine Leiomyoma	c.103_138del36	36	[4]
Uterine Leiomyoma	c.105_119del15	15	[4]
Uterine Leiomyoma	c.106_108del3	3	[4]
Uterine Leiomyoma	c.110_118del9	9	[4]
Uterine Leiomyoma	c.112_138del27	27	[4]
Uterine Leiomyoma	c.114_138del25insA	25ins1	[4]
Uterine Leiomyoma	c.115_141del27	27	[4]
Uterine Leiomyoma	c.116_122del7insC	7ins1	[4]
Uterine Leiomyoma	c.116_154del39	39	[4]
Uterine Leiomyoma	c.118_119insCGGCCTTGA	2ins9	[4]
Uterine Leiomyoma	c.121_138del18	18	[4]
Uterine Leiomyoma	c.122_157del36	36	[4]
Uterine Leiomyoma	c.123_134del12	12	[4]
Uterine Leiomyoma	c.126_140del15	15	[4]
Uterine Leiomyoma	c.128_145del18	18	[4]
Uterine Leiomyoma	c.129_131del3	3	[4]
Uterine Leiomyoma	c.133_150del18	18	[4]
Uterine Leiomyoma	c.138_152del15	15	[4]
Uterine Leiomyoma	c.141_161del21	21	[4]
Uterine Leiomyoma	IVS1-5_130del36	36	[4]
Uterine Leiomyoma	IVS1-5_137del43	43	[4]
Uterine Leiomyoma	IVS1-1_139del41	41	[4]
Uterine Leiomyoma	c.121_138del18	18	[7]
Leiomyosarcoma (metastasis, small intestine)	c.133_144del12	12	[7]
Uterine Leiomyoma	c.123_134del12	12	[5]
Uterine Leiomyoma	c.112_144delinsCTG	33ins3	[10]
Uterine Leiomyoma	c.126_143del	18	[10]
bizarre Leiomyoma	c.121_138del	18	[10]
Uterine Leiomyoma	IVS1-4_114del	19	[10]
Uterine Leiomyoma	c.126_134del9	9	[9]
STUMP	c.122_148del27	27	[9]
Uterine Leiomyoma	c.122_151del30	30	[11]
Uterine Leiomyoma	IVS1-4_138del43	43	[11]
Leiomyoma of the ovary	c.126_137del12	12	[11]
Leiomyoma of the kidney	c.110_136del27	27	[11]
Leiomyosarcoma of the retroperitoneum	c.133_144del12	12	[11]
Uterine Leiomyoma	c.109_117del9	9	unpublished data
Uterine Leiomyoma	c.110_118del9	9	unpublished data
Uterine Leiomyoma	c.118_136del19insC	19ins 1	unpublished data
Uterine Leiomyoma	c.120_122del3	3	unpublished data
Uterine Leiomyoma	c.120_122del3	3	unpublished data
Uterine Leiomyoma	c.120_125del6	6	unpublished data
Uterine Leiomyoma	c.120_125del6	6	unpublished data
Uterine Leiomyoma	c.122_148del27	27	unpublished data
Uterine Leiomyoma	c.122_160del39	39	unpublished data
Uterine Leiomyoma	c.123_152del30	30	unpublished data
Uterine Leiomyoma	c.125_160del36	36	unpublished data
Uterine Leiomyoma	c.125_160del36	36	unpublished data
Uterine Leiomyoma	c.126_131del6	6	unpublished data
Uterine Leiomyoma	c.126_140del15	15	unpublished data
Uterine Leiomyoma	c.126_143del18	18	unpublished data
Uterine Leiomyoma	c.133_150del18	18	unpublished data
Uterine Leiomyoma	c.135_149del15	15	unpublished data
Uterine Leiomyoma	c.136_150del15	15	unpublished data
Uterine Leiomyoma	c.146_166del21	21	unpublished data
Uterine Leiomyoma	c.147_161del15	15	unpublished data
Uterine Leiomyoma	c.131_136del6	6	unpublished data
Uterine Leiomyoma	IVS1-2_133del36	36	unpublished data
Uterine Leiomyoma	c.136_153del18	18	unpublished data
Uterine Leiomyoma	c.126_137del12	12	unpublished data

**Figure 1:**
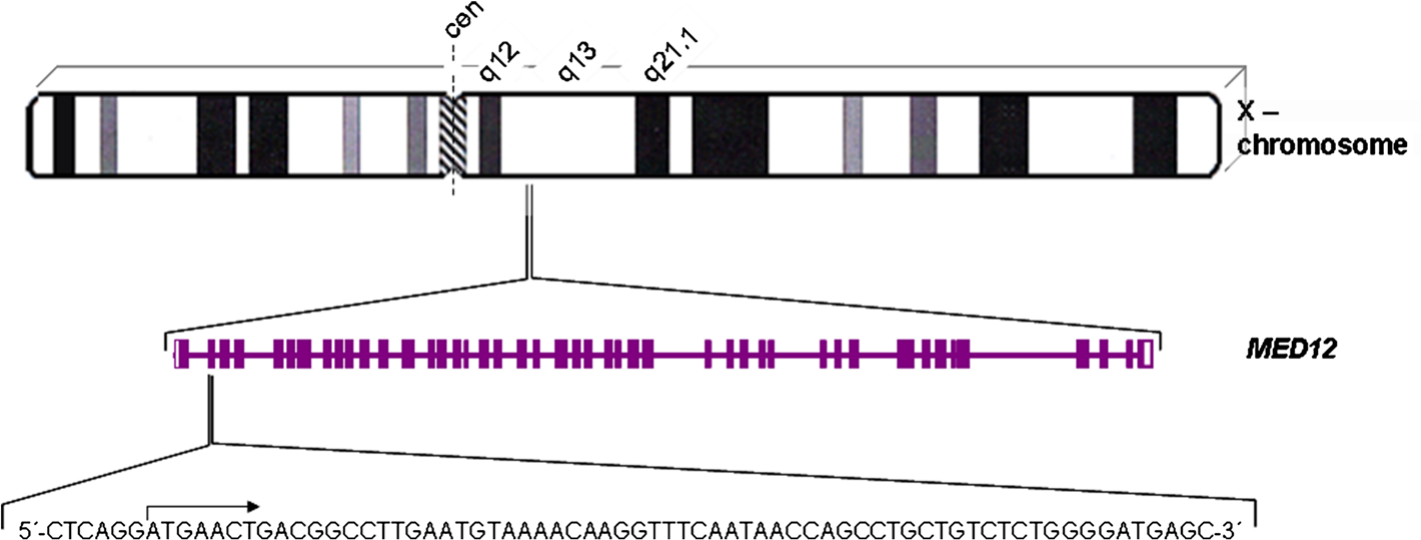
**Assignment of *MED12 *to Xq13.** The gene structure with exons (rectangles) and introns corresponds to the NCBI Map Viewer [12]. Bottom line: Sequence of the region affected by deletions. The start of exon 2 is indicated by an arrow.

**Figure 2:**
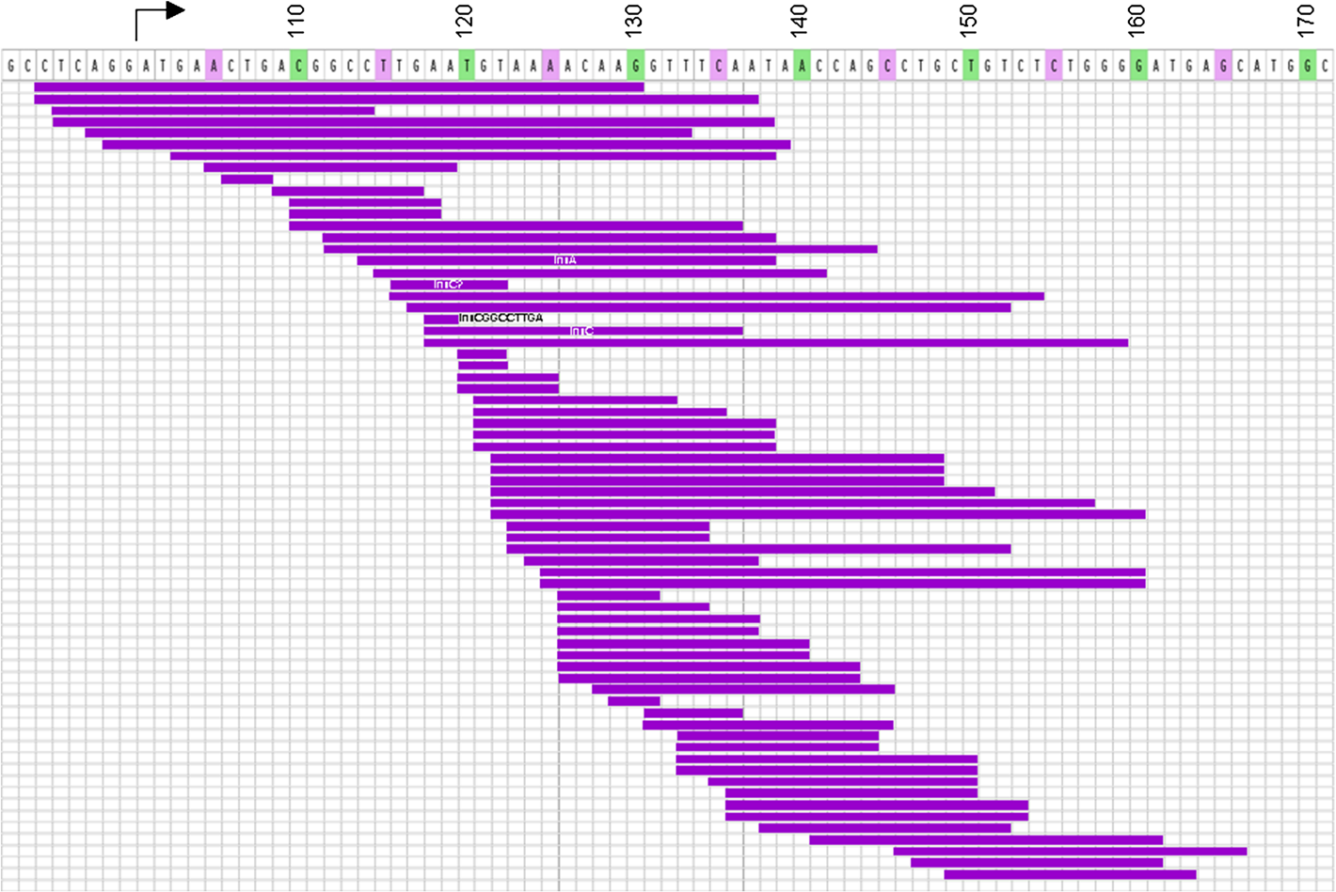
**Lengths and positions of 70 *MED12 *deletions from own unpublished cases and cases reported in the literature (c.f. Table**1**).** Each individual deletion seen in one smooth muscle tumor is given by a horizontal purple line. Bar on top of the illustration gives the segment affected by deletions in these tumors. Numbers correspond to nt. of the coding transcript.

In general, these *MED12* deletions covered a genomic region of 72 bp. At its 5’ end the region starts within intron 1 and ends at position c.166 of exon 2 (Figure 1). Within that deleted region the size of the individual deletions strongly varied in size ranging between 2 and 43 bp (Figure 2) with an average length of 18.7 bp. In five cases the deletions were accompanied by insertions but no insertions without deletions were observed (cf. Table 1).

**Figure 3:**
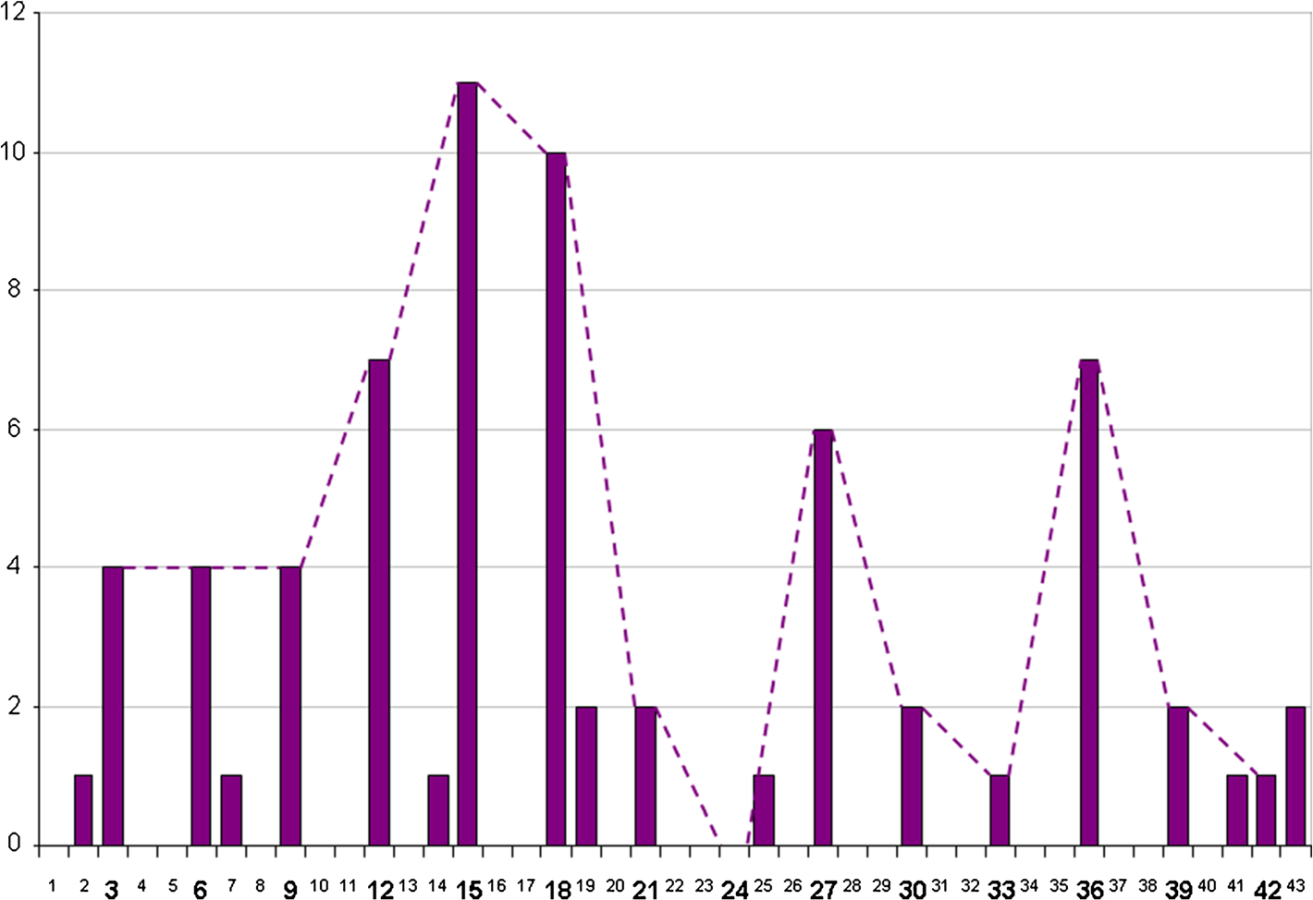
**Distribution of the lengths of the individual deletions highlighting a strong preference of multiples of 3 bp.** Dashed line links columns with multitudes of 3 to illustrate that among the latter cases those with deletions of 21, 24, 30, and 33 bp appear to be underrepresented.

As to the lengths of the individual deletions, a strong preference of multitudes of 3 is noted reflecting the fact that, as a rule, the deletions are gain of function mutations leaving the transcript in frame (Figure 3). Most of the lengths not representing multitudes of 3 were due to a start of the deletion within intron 1 with a presumed alteration of the splice site. Among the cases showing deletions with multitudes of 3 bp deletions of 21, 24, 30, and 33 bp were clearly underrepresented.

**Figure 4:**
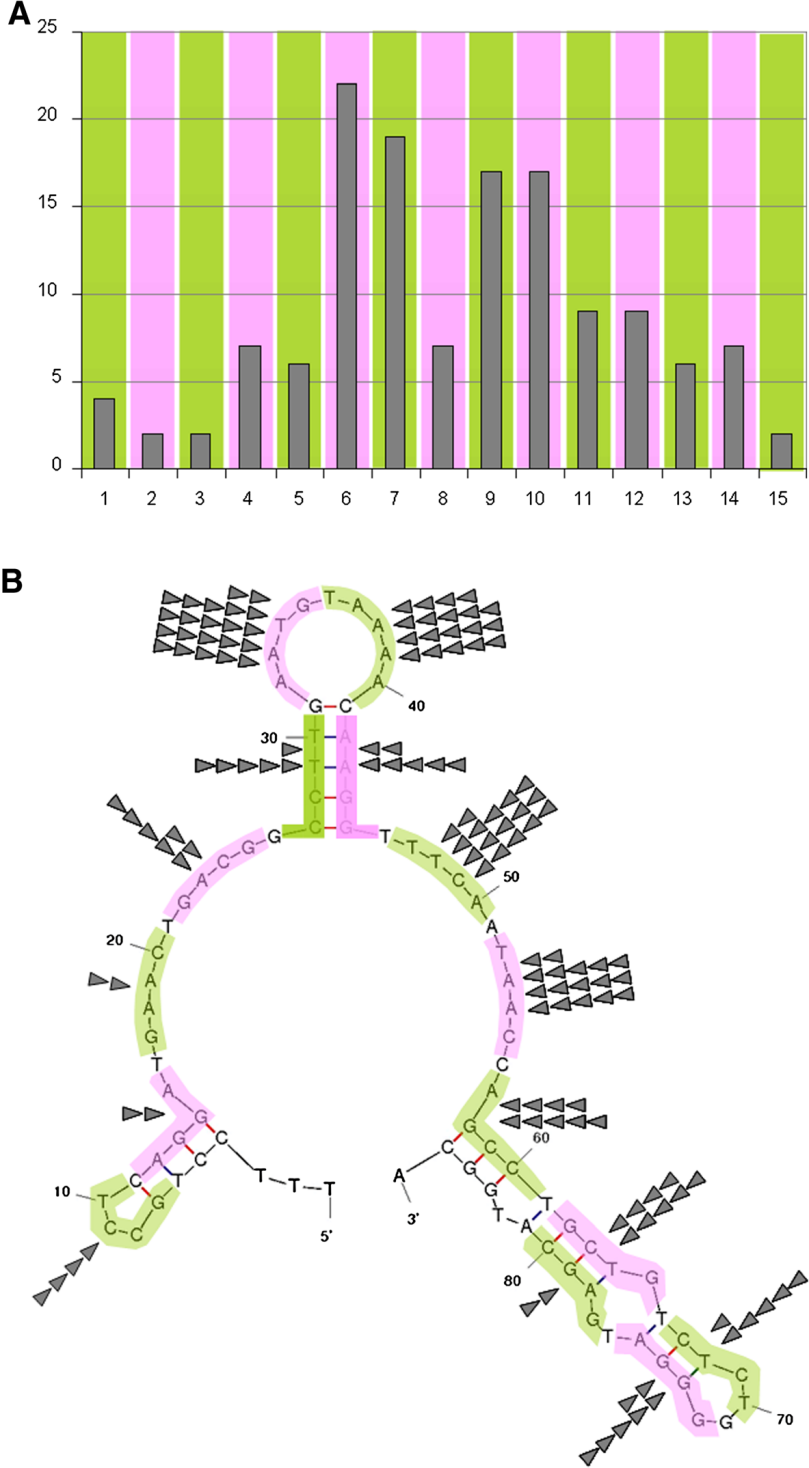
**A: For detailed analysis the *MED12 *segment affected by deletions was upstream and downstream extended by seven and six bp, respectively each and then subdivided into subsegments of five nt. each.** For illustration, these 5 nt. sliding windows are shown in pink and green, respectively. **B:** Folded structure of the DNA-segment covering the *MED12*-deletions seen in uterine smooth muscle tumors. For prediction the DNA segment affected by deletions (c.f. Figures 1 and 2) has been extended by seven and six nt., respectively, on both sides. Of the predicted structures by m-fold that with the lowest free energy (given as kcal/mol) is given. For analyses of non-randomly distribution, breakpoints within the five subsegment nt.-windows are corresponding to A. Breakpoints within each subsegment are given by triangles.

It seems reasonable to assume that the breakpoints are arranged over the whole region with their frequency more or less following a normal distribution. Accordingly, they indeed preferentially occurred in the center of the region. Nevertheless, surprisingly two peaks clearly became visible (Figure 4A). The reasons for this bimodal appearance are not clear but an influence of the local DNA structure is one of the likely explanations.

**Figure 5:**
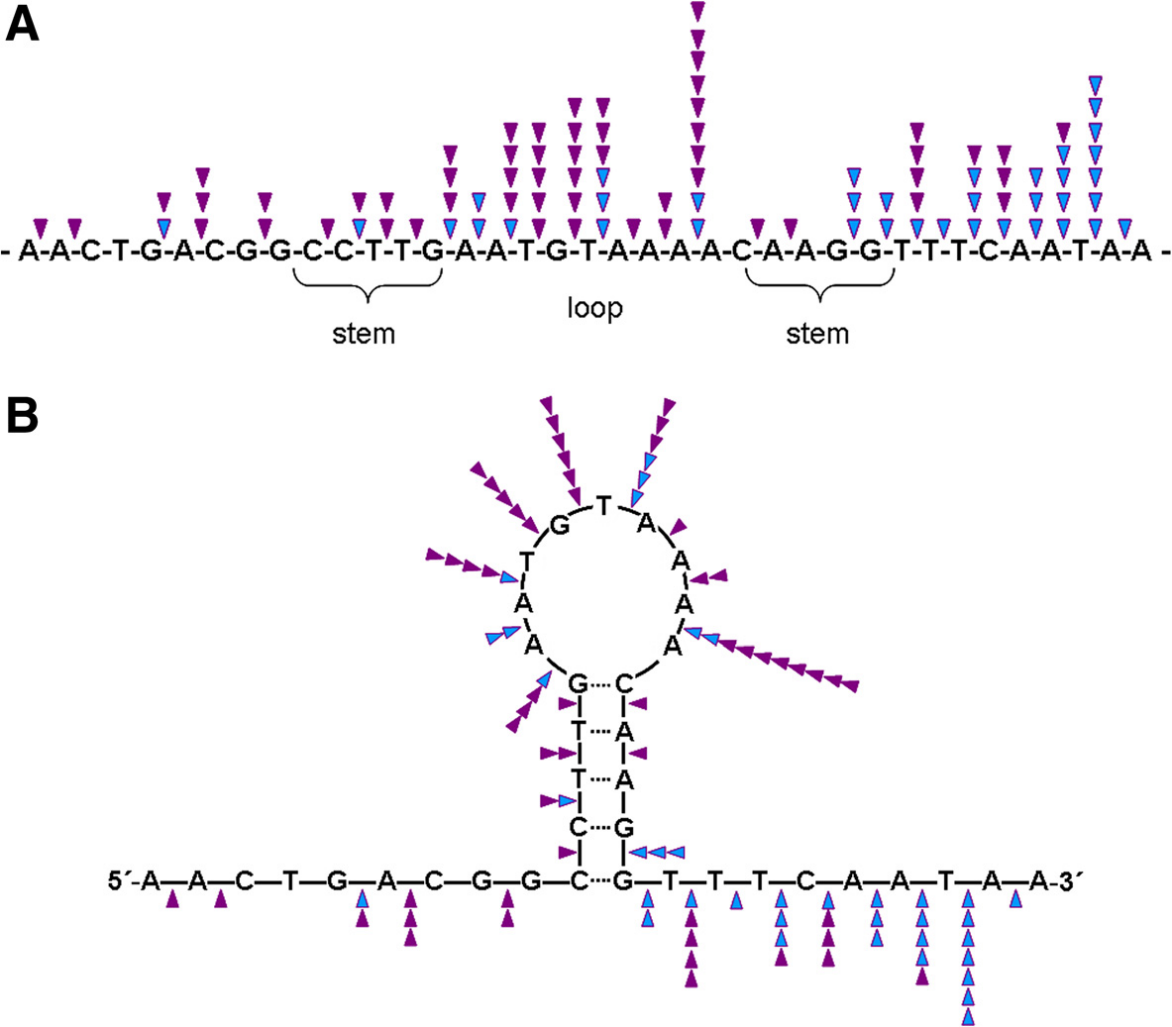
**Detailed analysis of the breakpoint distribution within and around the stem-loop structure predicted in the centre of the *MED12 *deletion DNA segment. A:** The DNA segment containing the central folded structure (c.f. Figure 4B) is given as a linear DNA strand. **B:** same segment as given in A shown as folded structure. Purple triangles: 5′ breaks, blue triangles: 3′ breaks.

Because non-canonical DNA conformations are considered as being a possible reason for deletions, we have used the m-fold software as well as the non-B DB search tool to predict hairpin structures in the part of *MED12* affected by the deletions. The non-B DB search tool did not predict non-B DNA structures for a DNA fragment of 86 bases extending the border of the affected segment by seven and six bp, respectively. In contrast, for the same segment the m-fold software predicted folded structures. Of these Figure 4B shows the one with the lowest free energy (-10.06 kcal/mol). Located almost in the center of the region affected by deletions a stem-loop structure due to a 6 bp inverted repeat exists that also covers the point mutation hotspot within codon 44 by its 3’stem region. Interestingly, the loop of this central putative hairpin structure is also a cluster region of deletion breakpoints. Another cluster with high frequency of breakpoints is the area downstream of the stem (Figure 4B) as also reflected by the bimodal distribution of the breakpoints (c.f. Figure 4A). An in depth analysis of this structure reveals a strong preference of the loop region to be affected by 5’ breaks and a preference of the region downstream of the stem for 3’breaks (Figure 5A and B).

## Discussion

The vast majority of uterine fibroids shows genetic alterations ranging from gross cytogenetically visible genomic deviations to point mutations. As to the type of mutations observed; large genomic deletions of parts of the long arm of chromosome 7 apparently constitute loss of function mutations [1] whereas in other cases genomic rearrangements like those of the *HMGA2* locus result in a gain of function. Another highly frequent type of mutations of the latter group are those affecting *MED12*[4-9,11] that are likely to constitute the most frequent gene alterations in human tumors at all. Quite recently, similar mutations were also observed in leiomyosarcomas and extrauterine leiomyomas [7,9-11,13] but they seem to be exceptionally rare in tumors not originating from smooth muscle tissue [13]. The almost exclusive presence of these mutations in smooth muscle tumors may point to some tissue specificity of the hotspot affected. Alternatively, it may reflect merely a growth advantage of affected cells in smooth muscle tissue.

In contrast to single base exchanges deletions within and around the hotspot region are only found in a minority of roughly 14.5% of the fibroids with *MED12* mutations. The c.130/131 hotspot for base exchanges is flanked by a much larger region where deletions of highly variable lengths can occur. These deletions clearly not only allow to delineate a hypermutable region of the genome but also represent even one of the most commonly deleted regions in human tumors known so far. In nearly all cases of “pure” exonic deletions multitudes of 3 bp had been removed which likely indicates clonal selection towards transcripts that remain in frame rather than a preferred primary occurrence of these sizes.

Constituting nearly 20%, microdeletions (≤ 20 bp) generally rank among the most common pathological human gene lesions [14]. Though most of the *MED12* deletions were not larger than 20 bp, these cases do not fit with the length distribution as reported for “typical” microdeletions in the literature: Germ-line microdeletions retrieved from the Human Gene Mutation Database (http://www.hgmd.org) have been analyzed by Ball et al. [14]: In general, a decreasing frequency of microdeletions with length was noted with more than 70% of the deletions ranging between 1 and 5 base pairs. In contrast, the lengths of the *MED12* deletions observed in smooth muscle tumors clustered between 2 and 43 bp and thus in many cases the length of the deletions even exceeded the range as defined for germline microdeletions (≤ 20 bp). These differences may point to different mechanisms favoring the occurrence of both types of deletions. As a mechanism to explain the “typical” microdeletions slippage; mutagenesis removing mono-, di- or trinucleotide repeats has been proposed [14,15]. In case of *MED12*, no evidence for a mechanism that simply removes short repeats has been obtained from the present analysis.

As to an alternative mechanism, it seems reasonable to speculate that the deletions are due to non-B DNA structures within the hotspot region. These structures like cruciform, triplex, quadruplex, hairpin, or left-handed Z-DNA conformations adopted at repeat sequences can favour e.g. the occurrence of mutations like microdeletions, microinsertions, and indels (for review see [16]) as well as chromosomal translocations [17]. In general, non-B DNA structures are considered as major determinants of genomic rearrangements in human diseases [18,19]. The pattern of breakpoint distribution over the affected region speaks in favour of such a hypothesis. We were able to show that one cluster of the deletion breakpoints is located within the center of a putative stem-loop structure with a second cluster downstream of the stem. In contrast, the sequences folding the intramolecular B-helix show a much lower frequency of deletion breakpoints. A similar clustering of deletion breakpoints within or near predicted non-B structures recently has been reported for deletions of human mitochondrial DNA [20]. Certainly, it cannot be ruled out that a deviation from the orthodox right-handed B-conformation or the association of the breakpoints with the folded structure does not explain the hypermutability of that *MED12* segment in smooth muscle tumors. On the other hand, the enormously high frequence of such deletions points to factors besides pure selection favouring this type of molecular alteration. Yet unknown links of folded DNA to its specific functions and behaviour as e.g. particular recombination systems might explain the patterns of *MED12* deletions as demonstrated herein. For prokaryotes, the participation of folded DNA in a number of processes is well documented (for review see [21]) and in eukaryotes as well, non-B DNA structures seem to serve, at least temporarily, important physiological functions. Leiomyomas are the most frequent symptomatic human tumors at all and the reasons for this frequent occurrence are not understood, yet. Naturally occurring non-canonical DNA structures have been shown to be prone to intrinsic mutagenicity in other types of mammalian tumors as e.g. Burkitt’s lymphoma and murine plasmocytomas [22,23]. In case of gross chromosomal rearrangements, Imagaki et al. [24] were able to elucidate the mechanisms by which such unusual DNA confirmations can be cut finally resulting in translocations, deletions, or inversions. To the best of our knowledge, the present study is the first attempt to trace back the highly frequent genetic alterations of *MED12* to particular DNA-structures. The non-canonical DNA structure itself as well as the mechanisms leading to breakage and reunion may be intrinsically driven or depend on unknown secondary factors. However, we feel that these initial data warrant further interest. In the future, experimental methods based on cloned fragments containing the deletion hotspot may allow more detailed analyses of the relevant mechanisms.

## Conclusions

Uterine fibroids are the most frequent symptomatic human tumors at all. Frequently, these tumors display mutations affecting the gene encoding mediator subcomplex 12 (*MED12*). In some of these cases *MED12* is affected by particular deletions major characteristics of which we have addressed in the present paper. Interestingly, the non-random distribution of breakpoints within the deletion hotspot region may point to a role of non-canonical DNA structures for the occurrence of these mutations and the molecular pathogenesis of uterine smooth muscle tumors, respectively.

## Methods

### Tumor samples

Of the *MED12* deletions analyzed herein 24/70 are from unpublished cases sequenced for *MED12* mutations. All samples were taken initially for diagnostic purposes and de-identified prior to their use in the present study following the rules of the Helsinki-declaration. Written informed consent was obtained from all patients and the study was approved by the local ethics committee (Ärztekammer Bremen; reference no #148). The remaining cases were taken from the literature (c.f. Table 1).

### DNA isolation

DNA from frozen tissue samples was isolated using the QIAamp DNA Mini Kit (Qiagen, Hilden, Germany) and DNA from FFPE tissue samples was isolated using the QIAamp DNA FFPE Tissue Kit (Qiagen) on a QIACube (Qiagen) according to the manufacturer’s instructions.

### PCR and sequencing

For PCR amplification 1000 ng of genomic template DNA were used. Primers to amplify the desired human PCR fragment of the genomic template DNA were those recently described [4]. Subsequently, PCR-products were separated by agarose gel-electrophoresis and the desired DNA-fragments/-bands were extracted by a QIAquick Gel Extraction Kit (Qiagen) using a QIACube (Qiagen) according to manufacturer’s instructions. DNA-sequencing of the purified PCR-products was performed by GATC Biotech (GATC Biotech, Konstanz, Germany).

### Analyses of DNA structures

For the analyses of DNA structures the m-fold web server software for DNA strands [25] was used. Folding was predicted according to the following parameters: folding temperature: 37°C, NA^+^: 1.0M, Mg^++^: 0.0M. Of the predicted structures that with the lowest free energy (ΔG, expressed as kcal/mol) was used for further analyses. In addition, for prediction of non-B DNA structures the non–B DB [26,27] search tool has been used.

## Competing interests

The authors declare that they have no competing interests.

## Authors’ contributions

DNM: conception and design of the study; acquisition of data; analysis and interpretation of data; manuscript writing. RN: analysis and interpretation of data. GB: analysis and interpretation of data. TL: provision of study material. BMH: provision of study material. JB: conception and design of the study; analysis and interpretation of data; manuscript writing; revising the manuscript critically for important intellectual content. All authors read and approved the final manuscript.
